# Supplementary material for: New Insights into Mucosa-Associated Microbiota in Paired Tumor and Non-Tumor Adjacent Mucosal Tissues in Colorectal Cancer Patients
Source: Cancers (Basel). 2024 Nov 29;16(23):4008. doi: 10.3390/cancers16234008 (PMC11640486; doi:10.3390/cancers16234008)
Supplement: Supplementary file 1 [file cancers-16-04008-s001.zip › cancers-3298359-supplementary.pdf]

Supplementary Table S1. Demographic and clinical data of samples.

**Data**	**CRC patients**
Age (mean, range)	65 (40-88)
Male/Female (%)	69.2/ 30.8
Tumor location (%)	
Colon	41.5
Rectum	43.1
Sigmoid colon	13.9
Cecum	1.5
Tumor differentiation (%)	
Well	7.7
Moderate	87.7
Poor	3.1
Undifferentiated	1.5
Tumor stage (%)	
0	1.5
1	6.2
2	26.2
3	49.2
4	7.7
Undetermined	9.2

Supplementary Table S2. Over-represented bacterial taxa in tumoral colon tissue compared to the rectum.

**Taxa name**	**Rank**	**Mean of colon relative abundance (%)**	**Mean of rectum relative abundance (%)**	**p-value**	**Log 2 Fold change**
Bacteroidota	phylum	18.87	16.5	$<10^{-6}$	0.14
Bacillota	phylum	24.39	20.02	$<10^{-6}$	0.26
Bacteroidia	class	12.58	11.03	$<10^{-6}$	0.14
Clostridia	class	20.03	16.94	$<10^{-6}$	0.26
Bacilli	class	4.2	3.279	$<10^{-6}$	0.38
Bacteroidales	order	12.95	11.34	$<10^{-6}$	0.14
Eubacteriales	order	15.6	13.19	$<10^{-6}$	0.26
Lactobacillales	order	4.47	3.56	$<10^{-6}$	0.38
Lachnospiraceae	family	8.34	6.83	$<10^{-6}$	0.26
Oscillospiraceae	family	3.40	2.68	$9.2*10^{-5}$	0.38
Prevotellaceae	family	2.82	1.90	$<10^{-6}$	0.58
Carnobacteriaceae	family	1.12	0.17	$<10^{-6}$	2.72
Prevotella	genus	2.65	1.71	$<10^{-6}$	0.58
Roseburia	genus	1.56	0.47	$<10^{-6}$	1.72
Granulicatella	genus	1.17	0.16	$<10^{-6}$	2.89
Leyella stercorea	species	1.76	0.99	$2.7*10^{-5}$	0.85

Agathobacter rectalis	species	1.11	0.31	$1.2*10^{-5}$	1.85
Phocaeicola plebeius	species	1.27	0.11	$<10^{-6}$	3.58
Granulicatella elegans	species	1.08	0.01	$<10^{-6}$	7.17

* Taxonomic name refers to the current name in the NCBI Taxonomy Browser (https://www.ncbi.nlm.nih.gov/Taxonomy/Browser/wwwtax.cgi).

Supplementary Table S3. Under-represented bacterial taxa in tumor colon tissue compared to the rectum.

Taxa name	Rank	Mean of colon relative abundance (%)	Mean of rectum relative abundance (%)	p-value	Log2 Fold Change
Fusobacteriota	Phylum	2.13	5.23	$<10^{-6}$	-1.29
Actinomycetota	Phylum	3.26	5.89	$<10^{-6}$	-0.86
Pseudomonadota	Phylum	5.86	6.99	$<10^{-6}$	-0.25
Fusobacteriia	Class	2.13	5.23	$<10^{-6}$	-1.29
Actinomycetes	Class	1.79	3.42	$<10^{-6}$	-0.94
Gammaproteobacteria	Class	2.52	3.28	$3.5*10^{-5}$	-0.38
Mycobacteriales	Order	0.51	1.60	$<10^{-6}$	-1.64
Fusobacteriales	Order	2.13	5.23	$<10^{-6}$	-1.29
Fusobacteriaceae	Family	1.08	3.47	$<10^{-6}$	-1.69
Rikenellaceae	Family	0.52	1.31	$1.9*10^{-5}$	-1.32
Propionibacteriaceae	Family	0.65	1.60	$<10^{-6}$	-1.29
Alistipes	Genus	0.52	1.29	$2.5*10^{-5}$	-1.32
Fusobacterium animalis	Species	0.37	1.11	$5.9*10^{-5}$	-1.56
Fusobacterium nucleatum	Species	0.68	3.89	$<10^{-6}$	-2.56
Fusobacterium polymorphum	Species	0.18	1.00	$7*10^{-6}$	-2.47
Fusobacterium vincentii	Species	0.10	1.14	$<10^{-6}$	-3.47
Streptococcus periodonticum	Species	1.02	2.57	$<10^{-6}$	-1.32

* Taxonomic name refers to the current name in the NCBI Taxonomy Browser (https://www.ncbi.nlm.nih.gov/Taxonomy/Browser/wwwtax.cgi).

Supplementary Table S4. Over-represented bacterial taxa in tumor colon tissue compared to sigmoid colon.

Taxa name	Rank	Mean of colon relative abundance (%)	Mean of sigmoid colon relative abundance (%)	p-value	Log2 Fold Change
Bacteroidota	Phylum	18.87	14.10	$<10^{-6}$	0.42

Bacteroidia	Class	12.58	9.39	$<10^{-6}$	0.42
Clostridia	Cass	20.03	14.77	$<10^{-6}$	0.44
Bacteroidales	Order	12.95	9.66	$<10^{-6}$	0.42
Eubacteriales	Order	15.60	11.50	$<10^{-6}$	0.44
Lachnospiraceae	Family	8.34	5.45	$<10^{-6}$	0.61
Prevotellaceae	Family	2.82	0.91	$<10^{-6}$	1.63
Roseburia	Genus	1.56	0.49	$5.1*10^{-5}$	1.66
Prevotella	Genus	2.65	0.83	$<10^{-6}$	1.67
Granulicatella	Genus	1.17	0.09	$4.5*10^{-5}$	3.63
Leyella stercorea	Species	1.76	0.12	$<10^{-6}$	3.87
Granulicatella elegans	Species	1.08	0.004	$4.4*10^{-5}$	8.12

* Taxonomic name refers to the current name in the NCBI Taxonomy Browser (https://www.ncbi.nlm.nih.gov/Taxonomy/Browser/wwwtax.cgi).

Supplementary Table S5. Under-represented bacterial taxa in tumor colon tissue compared to sigmoid colon.

**Taxa name**	**Rank**	**Mean of colon relative abundance (%)**	**Mean of sigmoid colon relative abundance (%)**	**p-value**	**Log2 Fold Change**
Actinomycetota	Phylum	3.26	6.24	$<10^{-6}$	-0.94
Pseudomonadota	Phylum	5.86	7.62	$<10^{-6}$	-0.38
Tissierellia	Class	0.60	1.98	$<10^{-6}$	-1.74
Actinomycetes	Class	1.79	3.67	$<10^{-6}$	-1.03
Gammaproteobacteria	Class	2.52	4.33	$<10^{-6}$	-0.79
Bacilli	Class	4.20	6.39	$<10^{-6}$	-0.60
Tissierellales	Order	0.71	2.38	$<10^{-6}$	-1.74
Lactobacillales	Order	4.47	5.96	$<10^{-6}$	-0.42
Staphylococcaceae	Family	0.22	1.60	$<10^{-6}$	-2.84
Peptoniphilaceae	Family	1.06	3.54	$<10^{-6}$	-1.74
Propionibacteriaceae	Family	0.65	1.75	$2.9*10^{-5}$	-1.43
Enterobacteriaceae	Family	0.79	1.88	$3.9*10^{-5}$	-1.25
Streptococcaceae	Family	2.55	5.47	$<10^{-6}$	-1.09
Peptoniphilus	Genus	0.05	1.56	$<10^{-6}$	-5.06
Staphylococcus	Genus	0.17	1.27	$3.1*10^{-5}$	-2.84
Streptococcus	Genus	2.59	5.57	$<10^{-6}$	-1.09
Fusobacterium	Genus	1.44	2.66	$4*10^{-6}$	-0.89
Fusobacterium nucleatum	Species	0.68	3.09	$<10^{-6}$	-2.18
Fusobacterium polymorphum	Species	0.42	2.13	$<10^{-6}$	-2.32

Fusobacterium vincentii	Species	0.10	1.68	$<10^{-6}$	-4.06
Peptoniphilus lacrimalis	Species	0.03	1.29	$2*10^{-6}$	-5.64
Phocaeicola coprocola	Species	0.17	1.21	$8.5*10^{-5}$	-2.84
Porphyromonas endodontalis	Species	0.02	1.06	$7.5*10^{-5}$	-6.64
Streptococcus periodonticum	Species	1.02	5.06	$<10^{-6}$	-2.32
Waltera intestinalis	Species	0.46	1.57	$2.3*10^{-5}$	-1.79

* Taxonomic name refers to the current name in the NCBI Taxonomy Browser (https://www.ncbi.nlm.nih.gov/Taxonomy/Browser/wwwtax.cgi).

Supplementary Table S6. Over-represented bacterial taxa in tumor rectum compared to sigmoid colon.

Taxa name	Rank	Mean of rectum relative abundance (%)	Mean of sigmoid colon relative abundance (%)	p-value	Log2 Fold Change
Bacteroidota	Phylum	16.50	14.10	$<10^{-6}$	0.23
Fusobacteriota	Phylum	5.23	2.92	$<10^{-6}$	0.84
Clostridia	Class	16.94	14.77	$<10^{-6}$	0.20
Bacteroidia	Class	11.03	9.39	$<10^{-6}$	0.23
Fusobacteriia	Class	5.23	2.92	$<10^{-6}$	0.84
Eubacteriales	Order	13.19	11.50	$<10^{-6}$	0.20
Bacteroidales	Order	11.34	9.66	$<10^{-6}$	0.23
Fusobacteriales	Order	5.23	2.92	$<10^{-6}$	0.84
Lachnospiraceae	Family	6.83	5.45	$<10^{-6}$	0.32
Fusobacteriaceae	Family	3.47	2.00	$<10^{-6}$	0.79
Fusobacterium	Genus	4.60	2.66	$<10^{-6}$	0.79
Fusobacterium animalis	Species	1.75	0.55	10^{-5}	1.68

* Taxonomic name refers to the current name in the NCBI Taxonomy Browser (https://www.ncbi.nlm.nih.gov/Taxonomy/Browser/wwwtax.cgi).

Supplementary Table S7. Under-represented bacterial taxa in tumor rectum compared to sigmoid colon.

**Taxa name**	**Rank**	**Mean of rectum relative abundance (%)**	**Mean of sigmoid colon relative abundance (%)**	**p-value**	**Log2 Fold Change**
Bacillota	Phylum	20.02	23.84	$<10^{-6}$	-0.25
Tissierellia	Class	0.80	1.98	$1.7*10^{-5}$	-1.29
Bacilli	Olass	3.28	6.39	$<10^{-6}$	-0.97
Tissierellales	Order	0.96	2.38	$<10^{-6}$	-1.29
Lactobacillales	Order	3.56	5.96	$<10^{-6}$	-0.74
Staphylococcaceae	Family	0.25	1.60	$<10^{-6}$	-2.74
Peptoniphilaceae	Family	1.44	3.54	$<10^{-6}$	-1.29
Streptococcaceae	Family	3.08	5.47	$<10^{-6}$	-0.84
Peptoniphilus	Genus	0.12	1.56	$<10^{-6}$	-3.84
Staphylococcus	Genus	0.19	1.27	$7.7*10^{-5}$	-2.74
Streptococcus	Genus	3.13	5.57	$<10^{-6}$	-0.84
Peptoniphilus lacrimalis	Species	0.003	1.29	$3*10^{-6}$	-8.97
Phocaeicola coprocola	Species	0.38	2.90	$<10^{-6}$	-2.94
Streptococcus periodonticum	Species	2.57	5.06	$<10^{-6}$	-0.97
Waltera intestinalis	Species	0.29	1.57	$3*10^{-5}$	-2.40

* Taxonomic name refers to the current name in the NCBI Taxonomy Browser (https://www.ncbi.nlm.nih.gov/Taxonomy/Browser/wwwtax.cgi).
